# Gender differences in subjective discontinuation symptoms associated with ketamine use

**DOI:** 10.1186/1747-597X-9-39

**Published:** 2014-09-22

**Authors:** Wen-Yin Chen, Ming-Chyi Huang, Shih-Ku Lin

**Affiliations:** Department of Addiction Science, Taipei City Hospital and Psychiatric Center, 309 Songde Road, Xinyi District, Taipei, 110 Taiwan; Department of Psychiatry, College of Medicine, Taipei Medical University, Taipei, Taiwan

**Keywords:** Ketamine, Epidemiology, Discontinuance symptoms, Gender difference

## Abstract

**Background:**

Recent substance abuse research indicates gender differences in the substance-related epidemiology, biological responses, progression to dependence, medical consequences and treatments. Studies exploring human sex-different responses to ketamine are rare and there has been no systemic survey of gender differences in ketamine use. Determining whether females are more susceptible than males to ketamine withdrawal symptoms and adverse effects is important, because it associated with treatment retention and outcome in drug users.

**Methods:**

The Taiwanese juridical system has implemented a new regulation on ketamine in the year 2009. Ketamine users who are caught by the police, are mandated to attend an educational program. We recruited ketamine offenders from February 2010 to May 2012 at the Kunming branch of the Taipei City Hospital, where the educational classes are held. A designed questionnaire was performed to gather information about demographic characteristics, discontinuation symptoms, concomitant use of other substances, and subjective experience of memory impairment or urinary discomforts, and to compare the gender differences.

**Results:**

A total of 1,614 ketamine users were surveyed and most of them were males (83.8%), with an average age of 26.3 ± 5.4 years. Female ketamine users presented significantly more discontinuation symptoms such as anxiety, dysphoria, and tremors compared with male users. 72.4% of total ketamine users smoked cigarettes concomitantly. Male ketamine users had a higher rate of concomitant betel nut use, while female ketamine users had a higher rate of concomitant hypnotic and alcohol use. 76% of total ketamine users reported cognitive impairment and 51.6% mentioned urinary symptoms. Furthermore, female ketamine users self-reported significantly greater levels of severity in cognitive impairment and urinary discomforts compared with male users. Less than 10% of total ketamine users in our study reported the desire to transfer for medical intervention or treatment, despite the high rates of discontinuation symptoms and negative physical side effects.

**Conclusions:**

Gender differences were noted in the subjective experience of discontinuation symptoms, concomitant substance use, and severity of impairment related to ketamine use. However, the probable cause of the gender differences found in this study requires further investigation. We hoped our study will stimulate further research in this field.

## Background

Ketamine is an anesthetic derivative of phencyclidine (PCP) with dissociative, analgesic and psychedelic properties. Although ketamine is classified as a hallucinogen, it is widely used in a variety of anesthetic procedures in pediatric, obstetric and geriatric patients [1]. Moreover, the antidepressant effects of ketamine in treating refractory depression were reported in recent years [2].

According to the latest systemic review by Morgan and Curran [3], there are now increasing concerns about the harmful physical and psychological consequences of repeated misuse of ketamine. Acute physical harms include risk of death from accidents, acute cardiac risk from poisoning; ketamine-induced chronic physical harms include ulcerative cystitis, renal dysfunction and ‘k-cramps’. Frequent, daily use is also associated with neurocognitive impairment, and most robustly, deficits in working and episodic memory [4]. Psychological effects of chronic ketamine use were related to depression and psychosis [5]. Ketamine withdrawal symptoms characterized by anxiety, shaking, sweating, palpitations and carvings seem to be key problems in frequent ketamine users and have been published by many case studies [6–8]. However, there has yet to be a coherent and agreed upon description of a specific ketamine withdrawal syndrome to guide treatment and diagnosis. A rapid development of tolerance was demonstrated in ketamine users, and many frequent users are concerned about addiction and report trying but failing to stop using ketamine. Ketamine dependence can lead to costly health services, which include the treatment of the addiction and its related impairments in social functions, as well as the chronic physical health problems that will likely incur follow-up costs across the lifespan.

Recreational use of ketamine was first documented on the west coast of the United States in the early 1970s [9]. Ketamine has not been a major drug of abuse within the past two decades, and only a few cases have been reported during this time [10, 11]. After the ecstasy epidemic, ketamine use has become more popular in recent years around the world, especially as a common club drug among the dance, rave, and squat party scenes. It is also becoming popular in the United Kingdom [12, 13], Australia [14], Hong Kong [15, 16], and Mainland China [17]. In the latest World Drug Report by United Nations Office on Drugs Control (UNODC) 2013 [18], Ketamine belong to new psychoactive substance (NPS) and not controlled by international drug conventions, but which may pose a public health threat. Part of ketamine’s growing popularity in South-East Asia may be due to its lower status in the regulatory systems and lower price point as a substitute for the increasingly expensive ‘ecstasy’ or methamphetamine.

Ketamine has also become a commonly abused substance in Taiwan during recent years, especially among youths and adolescents [19–21]. Evidence showed that most ketamine users in Taiwan also used ecstasy (MDMA) concomitantly. Detected rates of MDMA and ketamine were found to be as high as 76% and 47% respectively among rave party participants in Taiwan [20]. Leung et al. [22] conducted a focus group with club drug users and noted that there was a special drug use sequence widely practiced by Taiwanese poly-drug users. In a single drug use episode, MDMA was usually the first drug used, followed by ketamine and then marijuana. This unique sequence of polydrug use in a single episode is called “Trinity”.

Ketamine is scheduled as level III in the “Narcotic Harm Control Act” in Taiwan. Pure ketamine use is regarded as an offense and not a crime. One reason for the increase in the abuse of ketamine in recent years is the lack of restriction by authorities. Reports from the Ministry of Justice showed increase in both the amount of street ketamine seized by the police, as well as the number of offenders (namely drug dealers or traffickers). Around 3000 to 4000 offenders were caught by the police in Taiwan per month during the year 2012.

Recent substance abuse research indicates gender differences in the substance-related epidemiology, social factors, biological responses, progression to dependence, medical consequences, co-occurring psychiatric disorders, and barriers to treatment entry, retention, and completion [23]. For examples, in alcohol drinking, women’s drinking-related problems appear to progress more quickly than those of men [24], and women report more severe problems and experience more health-related consequences from substance use [25]. Stimulants may enhance sex differences in the dopamine system in limbic regions, which may partly mediate sex difference in drug abuse [26]. Craving and relapse of drug seeking in abstinent individuals have also been found to differ between men and women, and there is robust evidence that women are more likely to initiate drug misuse and relapse into drug use after withdrawal than men [27]. In ecstasy use, gender was found to significantly moderate the relationship between the consumption and design fluency [28]. When addicted to cocaine or amphetamine, women showed more impairment in decision making than men [29]. Studies exploring human sex-different responses to ketamine are rare. An animal study by Winters *et al*, who compared the differing analgesic effects of ketamine in male and female rats, indicated that females were much more sensitive to ketamine than males [30, 31]. Moreover, female rats showed a greater sensitivity to ketamine neurotoxicity and brain neural loss compared to males [32]. Determining whether females are more susceptible than males to ketamine withdrawal symptoms and adverse effects is important, especially in cognitive deficits, because it associated with treatment retention and outcome in drug users [33]. The aim of this study is to further the management strategy for the current epidemic of ketamine abuse in Taiwan. The researchers collected information on relevant demographic and clinical data, so as to analyze the pattern of ketamine use, the discontinuation symptoms, the progression of harm, users’ willingness to seek treatment, and gender differences among ketamine users in the Taipei metropolitan area.

## Methods

### Participants and procedures

In light of the increasingly severe problem of ketamine abuse, the Taiwanese juridical system implemented a new regulation that focused on ketamine users beginning in 2009. This new regulation stipulates that offenders caught by the police for using ketamine (as evidenced by urine screen results) will receive a fine ranging from 10,000 to 50,000 NT dollars and attend a mandatory educational class for 4-8 hours. The mandatory class presents an opportunity for the investigator to come into contact with ketamine users within a larger sample.

We recruited ketamine offenders from February 2010 to May 2012 at the Kunming branch of the Taipei City Hospital where the educational classes are held. Since we were not aware of established questionnaires to examine ketamine-related problems, we designed a questionnaire to include demographic characteristics, age, reasons for initial use of ketamine, likes and dislikes during ketamine use, experience of discontinuation symptoms related to ketamine, and current concomitant substance use. The survey contained additional questions regarding the degree of memory impairment and urinary discomforts. Information about reasons for ketamine use and users’ likes and dislikes during ketamine use was collected by open questions, while the remainder of the information was gathered through checklist questions. Since the Chinese version of the pelvic pain and urgency/frequency (PUF) questionnaire has been shown to be reliable and valid for assessment in patients with lower urinary tract symptoms associated with street-ketamine use [34], and the cut-off value of the total score range from 13-17 with detecting interstitial cystitis among different literature reviews [34, 35]. We divided items assessing for urinary discomforts into three groups: Nil, Mild, and Severe, to separate the servility among the ketamine users who may beyond the cut-off points. Nil indicates never experiences of the pelvic pain or urgency/frequency; Mild indicates symptoms such as urgency/frequency occasionally but not usually; Severe indicates symptoms such as both pelvic pain and urgency/frequency, or one of the symptoms achieve usually or always, or having received clinical helps. The severe group could indicate the total score above 11 in the PUF questionnaire. However, there was no known validated self-report scale to evaluate ketamine-related memory impairments. Therefore, we designed a self-report questionnaire that categorizes memory impairment into three levels of severity: Nil, Mild, and Severe. Nil indicates no memory impairments; Mild indicates slight memory impairments; Severe indicates obvious signs of memory decline.

During the educational class, the research assistant provided a comprehensive description of the questionnaire, and all subjects were informed that their legal status would not be influenced by whether or not they participated in the study. The procedure was confidential. Participants were not required to identify themselves unless they wished to leave their personal information for referral to further medical evaluation or intervention. The Institutional Review Board of Taipei City Hospital, which has been certificated by Forum for Ethical Review Committees in Asian and Western Pacific Region (FERCAP) has approved this study (TCHIRB-971212).

### Data analysis

Demographic and clinical characteristics of the patients were analyzed by Student *t*-test or Chi-square test. Statistical analyses were performed using SPSS version 12.0. Results were considered statistically significant at p < .05. age-adjusted associations between genders and risk of Logistic regression analysis was also conducted to assess ketamine-related cognitive impairment or urinary symptoms.

## Results

### Demographics

Of the 1624 ketamine offenders, 10 either refused or failed to complete the questionnaire. Therefore, our analysis included data from 1614 participants (Mean age: 26.3 ± 5.4 years; Male to female ratio: 5:1). Demographic characteristics are displayed in Table 1, with gender comparison. Most of them were males (83.3%), single (88.0%) and employed (83.9%). The average duration of ketamine use was about 4 years.

**Table 1:** **Demographics information and clinical data in ketamine users**

		Total	Male	Female	P value
Age, years ± SD (range)		26.3 ± 5.4 (15-54)	26.5 ± 5.5 (15-54)	25.0 ± 4.3 (18-38)	<0.001***
Gender, n (%)		1614	1353 (83.8%)	261(16.2%)	<0.001***
Marriage status (%)	Single	88.0	88.1	87.5	0.013*
	Married	11.2	11.1	12.1	
	Others	0.8	0.8	0.4	
Education, years ± SD		12.1 ± 2.3	12.2 ± 2.4	11.8 ± 2.1	0.056
Occupation	On job, n (%)	1354 (83.9)	1143 (84.5)	211(80.7)	
	Jobless, n (%)	252 (15.6)	202 (14.9)	50 (19.1)	
Age of first ketamine use, years ± SD		22.1 ± 5.7	22.3 ± 5.8	21.2 ± 4.8	0.001**
Other substance use (%)	Cigarette	72.4	73.9	64.8	0.002**
	Alcohol	16.5	15.7	20.7	0.049*
	Betel nut	15.8	18.4	2.3	<0.001***
	Hypnotics	4.7	2.7	15.3	<0.001***
	MDMA	4.4	4.1	5.7	0.246
	Meth-amphetamine	1.6	1.4	2.7	0.133
	Marijuana	1.5	1.6	1.1	0.622
	Heroin	0.5	0.5	0.4	0.777
	Glue	0.4	0.5	0.0	0.244

P value: difference between genders.

*p < 0.05, **p< 0.01, ***p< 0.001.

### Concomitant substance use and discontinuation symptoms

Among the ketamine offenders, 72.4% smoked cigarettes and 16.5% consumed alcohol. It was found that more male than female users used betel nut concomitantly. Also, female users used more hypnotics and alcohol than males. The prevalence of other concomitant substance use among ketamine users is also displayed in Table 1.

When stopping ketamine use, the most common discomfort mentioned by participants was fatigue (26.0%), followed by poor appetite (20.4%) and drowsiness (19.1%). Cravings, as well as anxiety and dysphoria, were common psychological symptoms at the discontinuation of ketamine use, especially in females. In addition, 3-5% of participants experienced physical symptoms such as palpitation, sweating or tremors during discontinuation. Gender differences in discontinuation symptoms are shown in Table 2. Female ketamine users presented with significantly more anxiety (23.4%), dysphoria (24.1%) and tremors (6.5%) compared with male users during ketamine discontinuation.

**Table 2:** **Subjective reports of symptoms during ketamine discontinuation**

Discontinuation symptoms	Total (%)	Male (%)	Female (%)	P-value
Fatigue	26.0	26.6	23.0	0.222
Poor appetite	20.4	20.7	18.8	0.481
Drowsiness	19.1	18.3	23.4	0.058
Craving	18.7	17.9	23.0	0.054
Anxiety	18.3	17.4	23.4	0.022*
Dysphoria	17.2	15.9	24.1	0.001**
Tremors	3.6	3.0	6.5	0.006**
Palpitation	5.0	4.5	7.3	0.059
Sweating	4.4	4.5	3.8	0.625
Great effort to resist use	4.0	3.9	4.6	0.609

P value: difference between genders.

*p < 0.05, **p< 0.01.

### Self-report about the cognitive impairment, urinary discomfort, and motivation to seek medical intervention

22.4% of total ketamine offenders reported that their cognitive ability was severely impaired by ketamine use, particularly in females (27.5%). In addition, more than half of the total participants had urinary symptoms. Compared with males, female ketamine offenders were more likely to develop severe cognitive impairment (OR, 95% CI =1.717, 1.075-2.741) and urinary symptoms (2.719, 1.501-4.928) according to their self-report. Gender differences in subjective reports of cognitive impairment and urinary tract symptoms among ketamine users are shown in Table 3.

**Table 3:** **Subjective cognitive impairment and urinary tract symptoms among ketamine users and the adjusted odds ratios (AORs) between genders**

		Total	Male	Female	AORs ^c^	P value	95% CI
Cognitive impairment	Nil	23.9%	24.7%	20.2%	reference	-	-
	mild	53.6%	53.9%	52.3%	1.275	0.258	0.837-1.942
	severe	22.4%	21.4%	27.5%	1.717	0.024*	1.075-2.741
Urinary tract symptoms	Nil	49.5%	49.7%	48.4%	reference	-	-
	mild^a^	45.8%	46.5%	41.9%	0.915	0.601	0.656-1.276
	severe^b^	4.8%	3.8%	9.7%	2.719	0.001**	1.501-4.928

^a^Indicating symptoms as urgency/frequency occasionally but not usually.

^b^Indicating symptoms as both pelvic pain and urgency/frequency, or one of the symptoms achieved usually or always, or having received clinical help.

^c^The reference category is Nil group in cognitive impairment and urinary tract symptoms respectively, after adjusted age.

P value: the risk of subjective cognitive impairment or urinary tract symptoms between genders 95% CI, 95% confidence intervals.

*p < 0.05, ** p< 0.01.

Despite 76% of ketamine users reporting cognitive impairment and 51.6% mentioning urinary symptoms, only 7% of ketamine users in our study opted to seek medical intervention or treatment under our transfer, with also statistically significant gender difference (male: 6.1%, female:11.7%; p = .001).

## Discussion

In this study, we explored the discontinuation symptoms of ketamine offenders in Taiwan, and examined the gender difference. The results demonstrated that female ketamine users had more severe self-report cognitive impairment and urinary symptoms related to ketamine use than male users. Our study is the first with a large sample size that showed a gender difference in the complications of ketamine use. The possible implication of our findings will be discussed.

### Discontinuation symptoms

Pharmacologically, ketamine’s main action is on glutamate, the major excitatory neurotransmitter in the brain. It is a non-competitive antagonist at one of the three glutamate receptors: the N-methyl d-aspartate (NMDA) receptor [3]. Ketamine also has less prominent actions at other receptor sites. It blocks muscarinic acetylcholine receptors and may potentiate the effects of gamma-aminobutyric acid (GABA) synaptic inhibition. In addition, ketamine induces activation of dopamine release [36] and acts as a weak agonist at μ opioid receptors [37]. Ketamine’s affinity to multiple receptors could theoretically explain its effects on cognition, mood, psychotic experiences and withdrawal symptoms. Even though most of the participants in this study are poly-drug users and the discontinuation symptoms reported here may have been the result of a combination of different substances, ketamine users related their symptoms to the discontinuation of ketamine in the questionnaire.

It is unclear if physical symptoms occur during ketamine withdrawal. However, the discontinuation symptoms mentioned in our questionnaire are quite similar to alcohol withdrawal. In fact, alcohol consumption was found to reduce certain ketamine discontinuation symptoms, such as anxiety, shaking, sweating, palpitation, and low mood [6]. Ketamine discontinuation can result in glutamate rebound with symptoms reminiscent of NMDA over-activity in alcohol withdrawal [6]. It is plausible that modified forms of alcohol withdrawal regimens can lessen symptoms of ketamine discontinuation.

### Gender difference

Gender differences were found in the participants’ subjective reports of discontinuation symptoms, as well as concomitant substance use and severity of impairment related to ketamine. In addition, female ketamine users reported a higher proportion of alcohol or hypnotic consumption compared to males. This tendency may be related to the significantly higher amounts of self-reported discontinuation symptoms such as anxiety and tremors in female ketamine users. In an animal study, it was suggested that the gonadal hormones in female rats played a critical role in enhancing the antidepressant-like effects of ketamine [38]. Therefore, we surmised that female ketamine users tended to experience dysphoric mood more frequently than males during ketamine discontinuation, as shown in our study.

A study about gender differences in abusers of amphetamine-type stimulants (ATS) and ketamine (K) in southwestern China [39] compared gender trends in ATS and ATS + K patients. For males, the ATS + K patients were more likely to develop psychotic disorders than ATS patients. For females, the ATS + K patients were more likely to develop cognitive impairment than ATS patients. Due to the central role of the NMDA-receptor in learning and memory, both acute and chronic use of ketamine exerts specific and wide-ranging effects on memory systems [4]. In line with previous animal study, female ketamine users were more vulnerable to cognitive deficits compared with males. On the other hand, a UK study showed an absence of gender difference in urinary symptoms among recreational ketamine users [40]. However, female ketamine offenders were more likely to develop severe urinary discomforts in our findings. This suggests that female ketamine users may experience a greater amount of impairment related to ketamine use, which may encourage them to seek medical intervention.

Biological mechanisms underlying female-specific ketamine effects are largely unknown. Acute administration of ketamine has been reported to decrease serum sex hormones including estradiol, progesterone, and testosterone in female rats [41]. It is possible that repeated ketamine may have specific effects on women via sex hormones; however, the probable cause of the gender differences found in this study requires further investigation.

### Limitation

Our results should be interpreted with some limitations in mind. First, since participants were recruited while being mandated by the law to attend the class, it was difficult to validate the actual frequency and amount of ketamine and concomitant substance use, and thus the data related to substance use should be underestimated. We designed the confidential self-report to have favorable ascertainment. Second, in the absence of established questionnaires to examine ketamine-related problems (e.g., discontinuation symptoms), the questionnaires we used have not been studied to determine reliability and validity. There was no definition of cognitive impairment in the questionnaire, so the severity may be inconsistent between individual. Third, the discontinuation symptoms reported in our study may have been the result of a combination of different substances; however, ketamine users related their symptoms to the discontinuation of ketamine in the questionnaire. Researchers should be cautious to generalize our results given the high comorbidity of substance use disorder with other mental health issues. In this study, neuropsychiatric disorders or current affective state were not identified. Previous studies showed that the lifetime rates of mood and anxiety disorders are significantly higher among women than men, with and without substance-use disorders [42]. Associations between most substance use disorders and independent mood and anxiety disorders were positive and significant [43]. It is important to conduct a more comprehensive psychiatric assessment to determine whether substance use may enhance vulnerability for these disorders, or lead to organic changes that manifest as a mood or anxiety disorder. Finally, there might be gender differences on how participants responded to the self-report questionnaires. Gender was an important demographic factor associated with symptom reporting, and most physical symptoms are typically reported more often by women than by men [44]. We cannot exclude the potential effects of gender on self-reporting biases in this study.

### Implications

Ketamine has had a turbulent history since its first use as a clinical anesthesia for humans in 1964 [45]. With its analgesic-anesthetic mechanisms and antidepressant effect, ketamine is associated with a high potential for abuse or dependence. The pros and cons of ketamine use, as well as other areas of uncertainty regarding its use (e.g. antidepressant effect), deserve further studies in the future.

Despite several limitations generalizing data derived from our study, experience has shown us that investigating subjects with substance use can be extremely difficult. This is the first article demonstrating the correlation between gender and ketamine-related problems. Our study demonstrated that female ketamine users had more severe self-reported cognitive impairment and urinary symptoms than male users. These findings were compatible with previous animal study. However, a lot of additional work is required for a better understanding of gender differences in ketamine use. We hoped our study will stimulate further research in this field. Currently, there are no guidelines for effective management of ketamine withdrawal effects or dependence [46]. As ketamine abuse and dependence grows, addiction services need to be better informed of the effects of this drug, especially among young females. Policies emphasizing the increased vulnerability to ketamine-related adverse effects in female users compared with male users and offering the opportunity to enhance medical accessibility may improve efforts to prevent progression of ketamine dependence. Further studies should aim to address the health problems experienced by this group and explore the best approach to treat ketamine addiction.

## Conclusions

Discontinuation symptoms and physical side-effects are not uncommon in these ketamine offenders. Gender differences were noted in the subjective experience of discontinuation symptoms, concomitant substance use, and severity of impairment related to ketamine use. Additional work is required for a better understanding of the gender differences found in this study. Ketamine users in Taiwan are generally relatively young; therefore, further studies should be conducted to increase the availability and accessibility of effective treatment interventions, and preventative public education.

## Abbreviations

PCP: Phencyclidine
UNODC: United Nations Office on Drugs Control
NPS: New psychoactive substance
MDMA: 3,4-methylenedioxy-N-methylamphetamine
NMDA: N-methyl d-aspartate
GABA: Gamma-aminobutyric acid.
